# In Vitro Rooting of Poplar: Effects and Metabolism of Dichlorprop Auxin Ester Prodrugs

**DOI:** 10.3390/plants14010108

**Published:** 2025-01-02

**Authors:** Hajer Darouez, Stefaan P. O. Werbrouck

**Affiliations:** Laboratory for Applied In Vitro Plant Biotechnology, Ghent University, 9000 Ghent, Belgium

**Keywords:** adventitious root induction, auxin prodrugs, aerial roots, dichlorprop, *Populus* × *canadensis*

## Abstract

Efficient adventitious root formation is essential in micropropagation. Auxin prodrugs, inactive precursors that convert into active auxins within the plant, offer potentially improved rooting control and reduced phytotoxicity. This study investigated the efficacy of dichlorprop ester (DCPE), commercialized as Corasil^®^ and Clemensgros^®^ (originally intended to increase grapefruit size), in promoting in vitro root initiation in the model plant *Populus* × *canadensis*, compared to its hydrolyzed form DCP and the related compound C77. DCPE displayed a stronger root-inducing effect than DCP, especially at lower concentrations (0.01 and 0.1 µM). Notably, at 1 µM, both DCP and DCPE induced abundant aerial root formation, a phenomenon not previously observed in poplar with traditional auxin treatments. Metabolite analysis revealed distinct patterns. DCPE treatment resulted in rapid hydrolysis to DCP, leading to faster and more systemic distribution of the active auxin throughout the plant, compared to direct DCP application. C77 treatments showed slower uptake and limited translocation combined with slow metabolism to DCP. These results highlight the potential of auxin prodrugs like DCPE as an effective and controllable auxin source for optimizing in vitro rooting protocols in woody plant species.

## 1. Introduction

**Figure 1:**
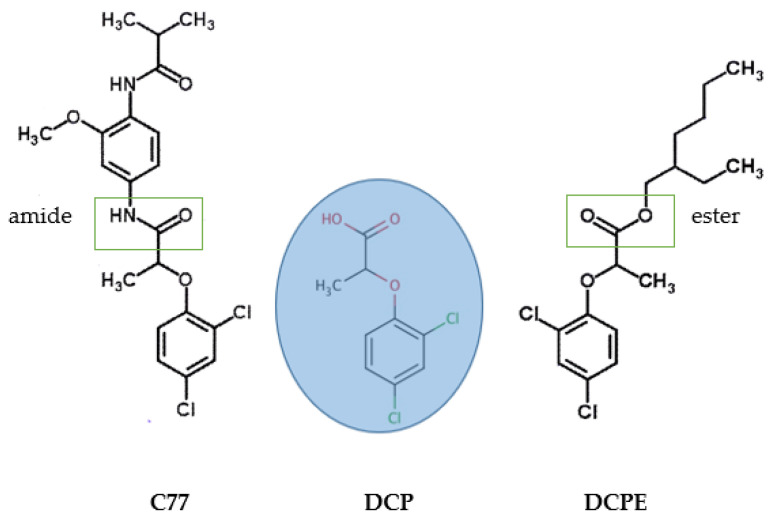
Chemical structures of C77 [22], DCP [23], and DCPE [23]. The greens squares indicate the amide group (left) and the ester group (right).

Auxin, a critical phytohormone, plays a key role in regulating plant growth, development, and responses to environmental signals. It coordinates essential processes such as organogenesis, pattern formation, and tropic growth by accumulating asymmetrically in tissues and organs [1,2,3,4,5]. These auxin functions are spatiotemporally regulated by specific pathways for biosynthesis, inactivation, transport, and signal transduction [6,7]. Auxin also plays an essential role in adventitious rooting, which is crucial for the vegetative propagation of economically important horticultural and woody species. While indole-3-acetic acid (IAA), indole-3-butyric acid (IBA), and 1-naphthaleneacetic acid (NAA) are commonly used auxins for inducing adventitious rooting in vitro, their effectiveness can be limited by factors such as chemical stability [8,9], metabolic conversion processes [10,11], hormonal balance within the plant [12], concentration levels used during treatment [13,14], and species-specific responses [15]. Bipartite auxin prodrugs represent an innovative approach to auxin delivery and action. These compounds are inactive precursors that are converted into active auxin molecules within the plant through metabolic processes, typically hydrolysis, by specific enzymes. Their key advantage lies in their potential for selective activation [16]. When esterases or amidases are expressed in specific tissues or developmental stages, targeted auxin responses can be achieved [17,18]. So far, research on auxin prodrugs has been limited to *Arabidopsis thaliana*. This is evident because the identification of auxin prodrugs was based on phenotype-based screening with chemical libraries. Some notable examples of this type of auxin prodrugs include WH7 [19], along with compounds 533 and 602 [20]. These molecules release phenoxy acetic acid-type auxins upon activation by plant amidohydrolases. They preferentially induce auxin responses in shoots, demonstrating tissue-specific activation. Sirtinol was initially identified as a synthetic activator of auxin signaling; however, it was later revealed to be a prodrug that is metabolized into the active auxin 2-hydroxy-1-naphthoic acid [21]. Particularly of interest for this research is the amide ester of Dichlorprop-P (DCP), compound 77 (C77). It was identified in a chemical genetic screen, notably as an anti-oxidative stress compound [22]. Dichlorprop-P, the (R)-(+) enantiomer of dichlorprop, is a versatile auxin-type phenoxy compound used in agriculture and horticulture, where it serves as a selective herbicide for controlling broadleaf weeds in cereals, pastures, and forestry. Its ester form, Dichlorprop-P-2-ethylhexyl (DCPE, Corasil), is commercially available as it is used as a plant growth regulator to increase fruit size and prevent premature fruit drop in citrus crops [23,24] (Figure 1).

To investigate the efficacy of C77 and DCPE in promoting adventitious root formation in recalcitrant woody species, we selected poplar as a model system for our in vitro rooting experiments. Optimizing these auxin prodrugs could enhance rooting efficiency in challenging woody species, thereby facilitating more effective vegetative propagation. Their distinctive properties may enable precise control over auxin delivery, allowing for the development of tailored rooting protocols for economically significant species in forestry and agriculture. It was hypothesized that both compounds would undergo enzymatic hydrolysis, releasing Dichlorprop-P (DCP) via deaminase and esterase activity, respectively, in a tissue-specific manner. As expected, the released DCP exerted abundant auxinic effects. To elucidate their mode of action, residue analyses were conducted to determine the absorption, metabolism, and distribution of these compounds within the in vitro plant tissues.

## 2. Results

### 2.1. Root Induction Through Continuous Application of Auxin Analogs

#### 2.1.1. DCP and DCPE

**Figure 2:**
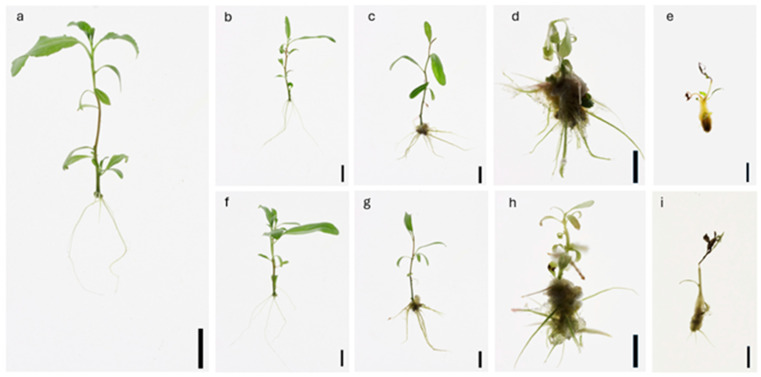
Root induction through continuous application of DCP and DCPE on *P. canadensis* after 25 days of in vitro culture (scale bar = 1 cm). The treatments included: (**a**) Control (hormone-free medium); (**b**) 0.01 µM DCPE; (**c**) 0.1 µM DCPE; (**d**) 1 µM DCPE; (**e**) 10 µM DCPE; (**f**) 0.01 µM DCP; (**g**) 0.1 µM DCP; (**h**) 1 µM DCP; (**i**) 10 µM DCP.

Figure 2 and Table 1 illustrate the distinct dose-dependent effects of DCP and DCPE on adventitious root development in poplar. Roots emerged in the control plants on day 4. However, in plants treated with DCP and DCPE, root emergence was observed as early as day 2 (Figure S1). DCPE, even at the lowest concentration (0.01 µM), induced a moderate increase in both the number and weight of adventitious roots, and promoted the growth of longer roots compared to the control, although a reduction in lateral root formation was observed. Conversely, at the same concentration, DCP did not affect the number and length of adventitious roots but did reduce root length and the number of adventitious roots, while substantially increasing root weight. At 0.1 µM, both compounds significantly enhanced the number of both adventitious and lateral roots, with DCPE exhibiting a stronger effect. However, this increased root induction was accompanied by a decrease in root length. Furthermore, DCPE exhibited a slightly greater propensity for callus induction at the shoot base compared to DCP.

**Figure 3:**
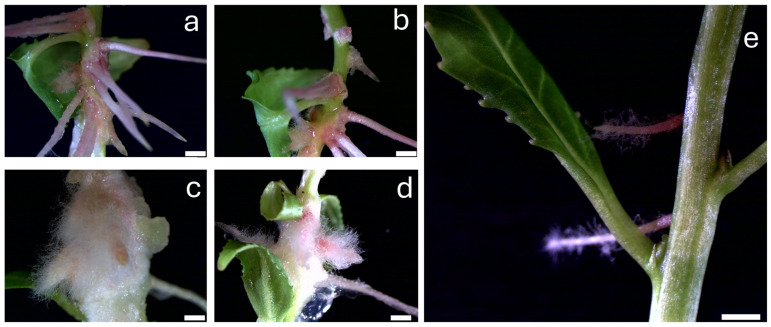
Rooting responses of *P. canadensis* to 1 µM DCPE (**a**,**c**), DCP (**b**,**d**), and C77 (**e**) after 8 days. (**a**,**b**) Middle stem sections. (**c**–**e**) Basal stem sections. Note the differences in root development and callus formation. Scale bar = 1 mm.

At a concentration of 1 µM, both DCP and DCPE exhibited a pronounced effect on rooting which made quantification impossible. As depicted in Figure 3, after 8 days, aerial root development was observed not only at the basal section but also in the middle sections of the plants. Roots induced by DCPE in the middle section were well developed, while those in the basal section were stunted, with a high density of root hairs and substantial callus formation (Figure 3a,c). Similarly, 1 µM DCP (Figure 3b,d) promoted adventitious root formation, but at a lower density compared to DCPE. Basal roots in DCP treated explants were finer and more dispersed, with less callus formation. However, after 25 days, intensive callus formation was observed, particularly in the DCPE-treated plants, where the callus was more pronounced compared to DCP. At 10 µM, the plants exhibited significant stress responses, including shoot tip necrosis and basal callus formation.

#### 2.1.2. C77

**Figure 4:**
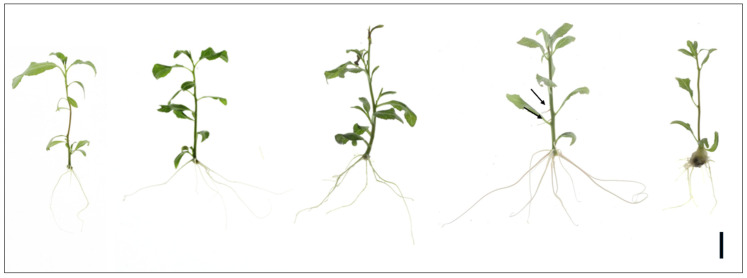
Effect of C77 on root induction in *P. canadensis*. Root development after 25 days of in vitro culture with increasing concentrations of C77 (from left to right: 0 µM, 0.01 µM, 0.1 µM, 1 µM, and 10 µM). Scale bar = 1 cm.

Similar to the control plants, root emergence in plants treated with C77 occurred on day 4 (Figure S2). As shown in Figure 4 and Table 2, C77 significantly influenced root development in *P. canadensis* after 25 days of in vitro culture. Low concentrations of C77 (0.01 µM and 0.1 µM) stimulated the formation of both adventitious and lateral roots, compared to the control. For example, 0.1 µM C77 resulted in an average of 6.2 adventitious roots and 23.11 lateral roots, exceeding the control values of 4.67 and 7.04, respectively. Treatment with 1 µM C77 resulted in an increased number of roots, but significantly reduced lateral root formation. Callus was not formed at this concentration. However, the highest concentration (10 µM) negatively impacted root development, significantly reducing adventitious root length to 1.03 cm, the shortest observed among all treatments. Root weight increased with increasing C77 concentrations, peaking at 7.6 mg with 1 µM C77 before declining at 10 µM. Notably, callus formation was observed exclusively at the highest C77 concentration (10 µM).

### 2.2. Distribution of DCPE, DCP, and C77 and Their Metabolite DCP

#### 2.2.1. DCP Metabolism over 8 Days

**Figure 5:**
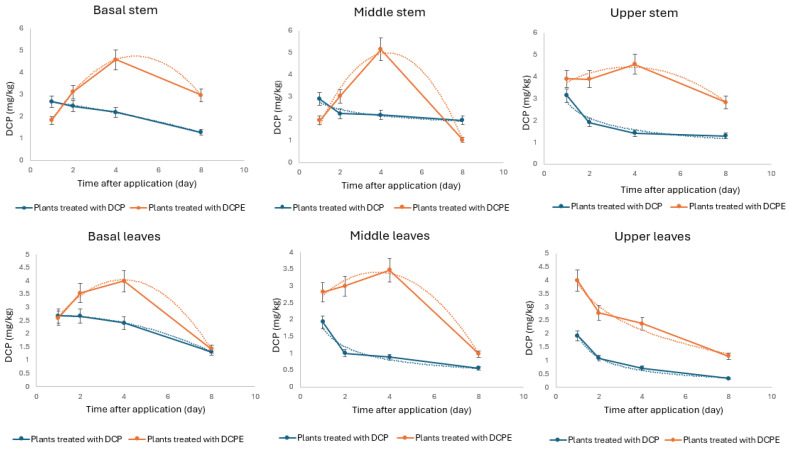
Dissipation of DCP in poplar stems and leaves over time after treatment with DCP and DCPE.

In *P. canadensis* treated directly with 1 µM DCP, the dissipation and metabolism of the compound exhibited distinct patterns across different plant sections (Figure 5). In the basal stems, DCP residues followed a quadratic trend, characterized by initial stabilization followed by a gradual decline, indicating efficient metabolism and dissipation. DCP levels in the middle stems displayed a power-law decay, with a slower, more gradual reduction over time. Similarly, DCP residues in the upper stems followed a power-law decay, marked by a consistent decline. In the leaves, DCP residues in the basal leaves also followed a quadratic model, with rapid reduction after a brief stabilization, suggesting effective transport and metabolism. DCP dissipation in the middle leaves exhibited a power-law decay, with a fast initial decline followed by slower decrease. The upper leaves similarly showed a power-law decay, characterized by a steady, gradual reduction in DCP levels over time (Table S1).

#### 2.2.2. DCPE Metabolism over 8 Days

Despite the addition of 1 µM DCPE to the growth medium, analysis of *P. canadensis* tissues revealed the absence of DCPE, while its hydrolyzed form, DCP, was detected. Figure 5 illustrates the temporal and spatial distribution of DCP within the plant. In the basal stem section, DCP levels increased over 4 days, then rapidly declined, exhibiting a quadratic relationship with time (Table S1). A similar quadratic trend was observed in the middle and upper stem sections. DCP concentrations in the basal and middle leaves also peaked around day 4, followed by a steady decline, best described by a quadratic model. In contrast, DCP levels in the upper leaves showed a rapid initial decrease followed by a slower decline, best fit by a logarithmic model (Table S1). This suggests rapid dissipation of the applied compound, with the initial peak likely corresponding to the time of application. These findings demonstrate that DCPE was rapidly absorbed and hydrolyzed within one day of entering the basal tissues of the poplar. Furthermore, the rate of DCP dissipation differed between the basal, middle, and upper regions of the plant (Figure 5).

#### 2.2.3. C77 Metabolism over 8 Days

**Figure 6:**
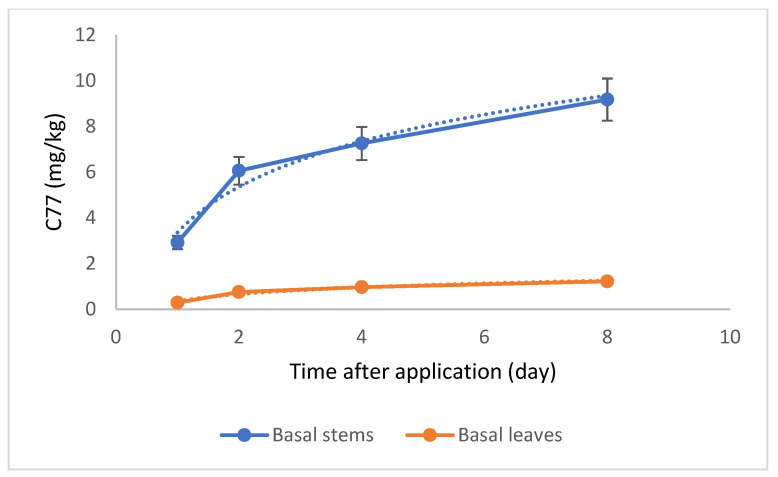
Dissipation of C77 in poplar basal stems and leaves over time after treatment with C77.

**Figure 7:**
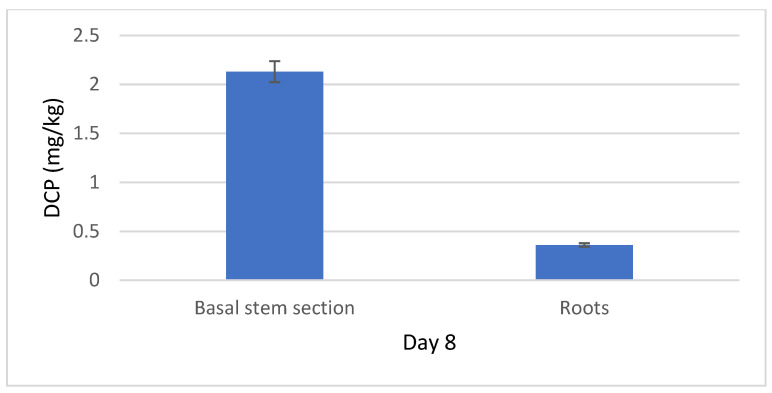
Dissipation of DCP in poplar stems and roots on day 8 after treatment with C77.

C77 residues were not detected in the middle and top sections of *P. canadensis*. However, C77 was present in the basal stem, exhibiting a logarithmic increase over time, rising from 2.92 mg/kg on day 1 to 9.17 mg/kg by day 8 (Figure 6). This logarithmic trend indicates a rapid accumulation during the initial four days, followed by a slower rate of increase. C77 was also detected in the leaves of the basal section, following a similar logarithmic increase, albeit at a much lower concentration than in the stems (Table S1). Notably, DCP was detected only on day 8, and only in the basal stem and roots, not in the leaves (Figure 7).

## 3. Discussion

In the present study, DCP and DCPE exhibited a pronounced effect on promoting adventitious root (AR) development in poplar explants, surpassing the efficacy of C77. At a concentration of 1 µM, both DCP and DCPE induced abundant aerial root formation, distributed across both the basal and middle sections of the explants. Interestingly, the basal aerial ARs were characterized by a swollen morphology and a dense covering of root hairs, while those emerging from the middle section maintained a normal appearance. In the top part of the shoot, there were no roots. The different effects of DCP and DCPE were most pronounced at the lowest concentrations, where DCPE-treated plants showed a greater abundance of roots with increased branching. The observed variation in rooting patterns cannot be solely attributed to differences in DCP concentration across the stem sections. Notably, the higher initial concentration in the apical section did not induce shoot formation. This suggests that other factors, such as differential tissue sensitivity to auxins, may play a significant role in determining rooting outcomes.

At higher concentrations (10 µM), both DCP and DCPE induced excessive callus formation and shoot necrosis, indicating a transition to herbicidal activity. This observation underscores the importance of careful dose optimization when using these compounds for rooting protocols. The application of synthetic auxin herbicides (SAHs), such as DCP, leads to an auxin overdose, disrupting the balance of endogenous auxin levels, which can either increase or decrease free indole-3-acetic acid (IAA) concentrations in plants [25]. For example, in *Galium aparine* treated with the synthetic auxin halauxifen-methyl, IAA levels rose significantly within 6 h and continued to increase for 24 h [26]. This auxin overload activates the production of ethylene and abscisic acid (ABA), with ethylene playing a key role in root formation. SAHs typically induce excessive auxin responses, triggering increased ethylene and ABA production [27,28,29,30]. Additionally, elevated IAA affects cellular processes like cell wall loosening and cell division, which are critical for root initiation [31].

Previous research into auxin prodrugs, particularly in *Arabidopsis*, has investigated compounds such as WH7 [19], 533 [20], and sirtinol [21], but none elicited the same profuse AR formation observed with DCPE in poplar. This highlights the importance of considering species-specific differences in auxin responses, as the mechanisms and efficacy of auxin prodrugs can differ significantly between herbaceous species like *Arabidopsis* and woody species such as poplar. Our study expands upon this knowledge, offering new insights into how DCP and DCPE can promote adventitious root formation in a woody species like poplar, an area that has been less explored in previous literature. DCPE was rapidly hydrolyzed to DCP. This is a selective, auxin-type phenoxy herbicide. This class includes other compounds such as 2,4-dichlorophenoxyacetic acid (2,4-D) and 2,4,5-trichlorophenoxyacetic acid (2,4,5-T). Both 2,4-D and 2,4,5-T have demonstrated limited efficacy in promoting root induction. While 2,4-D can stimulate growth at very low concentrations [32], just like DCP, it is phytotoxic at higher concentrations. Furthermore, both compounds induce callus formation in in vitro explants, a characteristic that can be exploited for somatic embryogenesis [33]. Although DCP is a synthetic auxin analog and primarily recognized for its herbicidal properties, its 2-ethylhexyl ester form (DCPE) is utilized as a plant growth regulator to enhance citrus fruit size and was reported to, directly or indirectly by releasing DCP, stimulate primary and lateral root formation in *Arabidopsis* [22].

The inability to detect DCPE in poplar suggests that it is almost completely converted to DCP soon after entering the plant. This emphasizes that the observed physiological effects, such as widespread rooting, are primarily attributed to the local activity of DCP. While DCPE plays a crucial role in delivery, it is ultimately the DCP that interacts with the plant’s auxin signaling pathways to trigger the developmental responses.

Despite employing various established auxin treatments, including 10 µM indole-3-butyric acid (IBA), 1-naphthaleneacetic acid (NAA), or 2,4-dichlorophenoxyacetic acid (2,4-D), we were unable to replicate this effect in our poplar explants. This suggests that C77, but especially DCP and DCPE, may operate through a unique mechanism or pathway in stimulating AR development in this species. In comparison, the application of C77 stimulated AR development in a dose-dependent manner, with a noticeable effect observed even at a concentration of 0.01 µM. This stimulatory effect persisted up to a concentration of 1 µM, at which point the formation of aerial ARs was observed. However, a further increase in concentration to 10 µM did not result in any additional increase in the basal AR count; instead, the ARs at this concentration exhibited a shorter morphology and were gradually overgrown by callus tissue. While active auxins are known to stimulate adventitious root initiation, they can also exert an inhibitory effect on root elongation [19]. In our study with poplar, both DCP and DCPE demonstrated a pronounced inhibitory effect on root elongation, with a noticeable reduction in root length observed at concentrations of 1 µM and above. In contrast, C77 only exhibited root growth inhibition at a higher concentration of 10 µM after 25 days of exposure. Regarding lateral roots, C77 exhibits a maximum number of lateral roots at a concentration of 0.1 µM. DCPE as well as DCP also demonstrate this stimulatory effect.

To investigate the uptake and metabolism of the compounds, a concentration of 1 µM was selected, as this concentration elicited the most pronounced root and callus induction. Due to its reduced overall polarity, the 2-ethylhexyl ester of dichlorprop (DCPE) exhibits inherently greater lipophilicity compared to its acid form, dichlorprop (DCP) [34]. This enhanced lipophilicity facilitates rapid penetration of the waxy cuticle of in vitro shoots, a characteristic that was intentionally designed to improve plant uptake. However quickly DCPE enters the plant, it appears to be hydrolyzed just as quickly in the plant, as it was not detected in its intact form after 24 h. DCPE’s prodrug nature is similar to that of many pharmaceutical compounds, where modifications improve bioavailability and therapeutic efficacy. Upon absorption, DCPE undergoes enzymatic hydrolysis to release DCP, which then regulates growth processes as an auxin analog. This prodrug system enhances solubility and stability, ensuring a controlled release of the active compound, minimizing potential toxicity at the absorption site [35,36].

Furthermore, few data are available on DCPE safety and metabolism in the plant. Álvarez et al. [23] investigated the metabolism of [^14^C] ring-labeled DCPE in field-grown wheat and orange trees. Analysis of mature wheat straw revealed the absence of detectable DCPE. The primary identified component was dichlorprop-P (DCP-P) (18.7%), followed by the breakdown products 2,4-dichlorophenol (1.8%) and 2-(2,5-dichloro-4-hydroxyphenoxy)propanoic acid (5.3%). A substantial proportion (14.4%) of the metabolites were identified as glycosides, suggesting that conjugation with sugars is a key detoxification and storage mechanism for DCP-P in wheat. Furthermore, the presence of dichlorprop-P methyl ester (14.1%) indicates that methylation constitutes another metabolic pathway. In contrast to wheat, only DCPE and DCP were detected in orange trees, while solely DCP was found in poplar. These species-specific differences in DCPE metabolism highlight the importance of studying auxin prodrug metabolism across different plant species and support our findings of rapid DCPE hydrolysis to DCP in poplar. Future research should include comprehensive metabolomics analysis to track the conversion of DCPE to DCP and any intermediate metabolites, as well as enzymatic assays to identify and characterize the specific esterases responsible for DCPE hydrolysis in poplar tissues.

Thanks to this fast hydrolysis, the continuous uptake of DCPE from the media acts like a slow-release mechanism for DCP. This means that the DCPE-treated plant has a rapid and constant supply of DCPE being converted to DCP, leading to higher overall levels and early rooting. DCP, whether applied directly or formed from DCPE, is transported rapidly and accumulates in stem and leaves. This targeted accumulation could result in higher DCP concentrations in those tissues in the DCPE-treated plants due to the continuous supply from DCPE hydrolysis [37]. In plants treated with DCP, the exogenous supply resulted in a comparable initial concentration as in DCPE-treated plants, resulting in similar fast rooting. But its supply appears insufficient to compensate for the rate of DCP degradation, resulting in a consistent decline in DCP concentration across all stem and leaf tissues. Conversely, in DCPE-treated plants, the continuous hydrolysis of DCPE provides a sustained source of DCP, potentially exceeding the plant’s metabolic capacity and resulting in transiently elevated DCP concentration that later declines following a quadratic trend.

In contrast to DCPE, C77 demonstrates greater stability within the plant, suggesting a longer persistence before being metabolized into DCP. This increased stability is likely due to the greater resistance of the amide bond in C77 to hydrolysis compared to the ester bond in DCPE [11]. DCP was first detected on day 8, exclusively in the basal stem and roots, suggesting a slow metabolism of C77 into its auxin-active form. This delayed conversion likely reduces auxin availability during critical developmental stages, potentially accounting for the reduced rooting responses observed with C77 compared to DCPE, which rapidly hydrolyzes into DCP upon absorption. C77’s limited translocation, slow metabolism, and delayed conversion to DCP likely result in insufficient auxin activity in the middle part of the explants, hindering root formation. Further research into the molecular mechanisms of C77’s action is needed to determine whether it exhibits auxin-like activity prior to hydrolysis. These findings underscore the complex interplay between prodrug stability, metabolic conversion, and physiological responses in plant development.

## 4. Materials and Methods

*Populus* × *canadensis* was micropropagated from foliated nodal segments (approximately 1.5 cm) on Quoirin and Lepoivre (QL) medium [38] containing macro- and micronutrients, vitamins, 3% sucrose, and 0.7% plant agar (basal medium). The medium was adjusted to pH 5.8, autoclaved (121 °C for 20 min), and supplemented with 5 µM mTR. Cultures were grown at 25 °C ± 1 °C under a 16/8 h light/dark photoperiod with warm white fluorescent light (40 W LED panel [Ledstores Europe, Amsterdam, The Netherlands]; 3000 K; 40 µmol m^−2^ s^−1^ PAR). Subculturing was performed every six weeks.

Internodal stem segments (approximately 1.5 cm) obtained from the shoot cultures were transferred into 380 mL glass containers on basal medium supplemented with 0, 0.01, 0.1, 1, or 10 µM of DCP, DCPE, and C77. The experiment used a completely randomized design (3 compounds × 5 concentrations per compound × 3 recipients per compound and concentration × 6 shoots per recipient) and was repeated three times. Cultures were maintained at 25 ± 1 °C under a 16/8 h light/dark photoperiod. After 25 days, the number of adventitious and lateral roots, average adventitious root length, and root and callus weight were recorded. Statistical analysis was conducted using IBM SPSS Statistics for Windows, version 29 (IBM Corp, North Castle, NY, USA) with a significance level of 5% (α = 0.05). Data normality was assessed using the Kolmogorov–Smirnov test. For all data, the non-parametric Kruskal–Wallis H test was employed (α = 0.05) to evaluate the differences between groups. In this case, the alternative hypothesis (H1) was retained.

**Figure 8:**
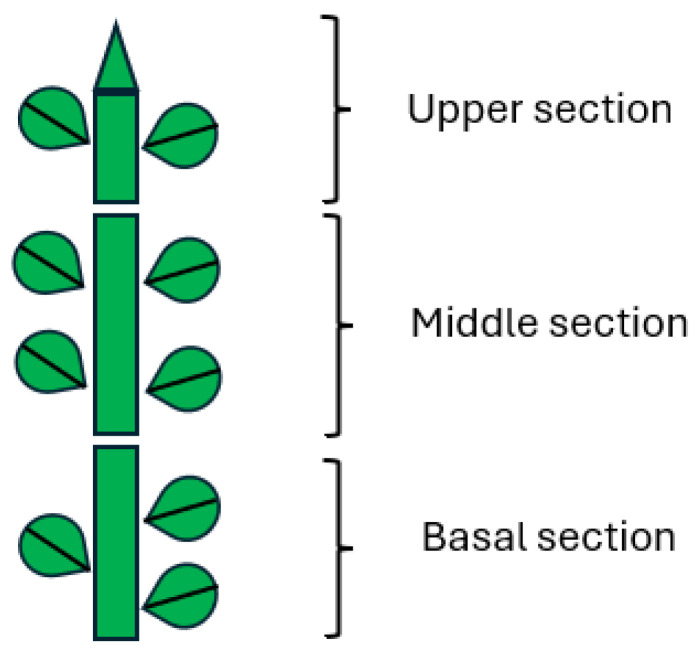
Different plant sections used for DCP, DCPE, and C77 quantification.

Foliated shoots (approximately 3 cm) from stock cultures of *P. canadensis* were transferred in groups of six to glass jars containing basal media supplemented with 1 µM of DCP, DCPE, or C77. DCPE was applied as Corasil (25 g/L Dichlorprop-P 2 Ethyl Hexyl Ester), 3 × 6 individual shoots were harvested at 0, 1, 2, 4, and 8 days. Root samples were also collected on day 8. Each shoot was divided into three sections (basal, middle, and top), and stem and leaf tissues were separated (Figure 8). This process was repeated three times. For each compound and time point, the six fractions from the 3 × 18 shoots were pooled, resulting in three biological replicates. Samples were lyophilized and stored at −20 °C for one month until analysis.

Frozen samples were thawed at room temperature and weighed into 50 mL centrifuge tubes. Extraction was performed by adding 2.5 mL of 0.1% (*v*/*v*) formic acid in water, 5 mL of acetonitrile, and 1.5 g of NaCl to each tube. Samples were vortexed for 2 min and centrifuged at 3800 rpm for 5 min. The supernatant (4 mL) was evaporated, and the residue was dissolved in 2 mL of 1:9 (*v*/*v*) acetonitrile: water. Extracts were stored at −21 °C until LC-MS/MS analysis.

LC-MS/MS analysis and quality validation procedures were performed as described in Houbraken et al. [39]. The product extracts were analyzed on a Waters ACQUITY UPLC™ system, equipped with a quaternary pump and membrane degasser. The separation column, an Acquity UPLC BEH C18, 130 Å, 1.7 µm, 2.1 mm × 50 mm, was kept at 40 °C. An automatic injector was set to inject 10 µL per sample. The mobile phase components, purchased from Sigma-Aldrich, were (A) Milli-Q water with 0.1% formic acid and (B) acetonitrile. The gradient used was set at a flow rate of 0.4 mL min^−1^ of 98% mobile phase A for 0.25 min. From 0.25 min to 7 min, a linear gradient was used to 98% mobile phase B, which was maintained for 1 min. Then, a linear gradient was used to return to 98% mobile phase A, and this was maintained for 1 min.

Sample analyses were performed using a triple quadrupole system (tandem MS-MS) with electrospray ionization (Waters Xevo^®^ TQD mass spectrometer, Milford, Massachusetts, USA). The capillary needle was maintained at +2 kV. For operation in the MS/MS mode, the following parameters were set: curtain gas (N_2_) at 7 bar; temperature 500 °C. The active ingredients of pesticides were monitored and quantified using multiple reaction monitoring (MRM). The recovery analysis was conducted using the spike-placebo recovery method. For this, eight blank samples were spiked with DCP and analyzed under the same conditions with the same extraction procedure. As the concentration and volume of the spiked solution were known, the recovery could be calculated. The resulting concentrations were converted to initial concentrations, and concentrations below the limit of detection (LOD) and the limit of quantification (LOQ) were filtered out. The final results were adjusted for each active ingredient and reported as the concentration of the detected compound residues in the poplar plant.

## 5. Conclusions

This study has highlighted the potential of auxin ester prodrugs as a means of controlling the release of active auxins for in vitro root induction. In particular, the commercially available DCPE (Corasil^®^ or Clemensgros^®^) shows promise for replacing traditional auxin treatments. Its rapid uptake and hydrolysis, leading to a sustained release of DCP, resulted in faster and more prolific root development compared to direct DCP application or the prodrug C77. The widespread availability of DCPE, originally intended for enhancing fruit size in grapefruits, could offer a cost-effective alternative to expensive, custom-synthesized prodrugs like C77.

The unique rooting patterns induced by DCP and DCPE, particularly at 1 µM concentrations, suggest novel auxin mechanisms in woody species. However, phytotoxicity at higher concentrations highlights the need for careful protocol optimization for use in recalcitrant woody species. Short-term or “pulse” applications could provide more precise control over DCP release, balancing root initiation with limiting excessive root and callus formation. Understanding how auxin prodrugs like DCPE and C77 influence root development in a model species such as poplar could contribute to broader advancements in woody plant propagation and genetic engineering.

## Figures and Tables

**Table 1:** Effect of DCPE and DCP on the number of adventitious and lateral roots, average adventitious root length (cm), and root and callus weights (mg) in *P. canadensis* after 25 days of in vitro culture.

Auxin Prodrug Treatments	Adventitious Root Number	Lateral Root Number	Average Adventitious Root Length (cm)	Root Weight (mg)	Callus Weight (mg)
Control	4.67 ± 0.34 ^a^	7.04 ± 0.67 ^b^	2.31 ± 0.09 ^a^	3.41 ± 0.85 ^a^	0 ± 0 ^a^
0.01 µM DCPE	6.2 ± 0.39 ^b^	5.22 ± 0.66 ^a^	3.28 ± 1.8 ^b^	5.46 ± 0.64 ^b^	0 ± 0 ^a^
0.1 µM DCPE	15.83 ± 0.7 ^d^	22.35 ± 1.2 ^d^	1.72 ± 0.1 ^c^	5.21 ± 0.79 ^b^	0.38 ± 0.034 ^c^
0.01 µM DCP	4.3 ± 0.28 ^a^	5.15 ± 0.5 ^a^	2.79 ± 0.18 ^ab^	7.6 ± 0.77 ^c^	0 ± 0 ^a^
0.1 µM DCP	13.7 ± 0.68 ^c^	18.44 ± 1.02 ^c^	1.43 ± 0.09 ^c^	2.89 ± 0.32 ^a^	0.23 ± 0.02 ^b^

Averages ± SE followed by the same letter in the same column are not significantly different at *p* < 0.05 according to the Kruskal–Wallis H test.

**Table 2:** Effect of C77 on the number of adventitious and lateral roots, average adventitious root length (cm), and root and callus weights (mg) in *P. canadensis* after 25 days of in vitro culture.

Auxin Prodrug Treatments	Adventitious Root Number	Lateral Root Number	Average Adventitious Root Length (cm)	Root Weight (mg)	Callus Weight (mg)
Control	4.67 ± 0.34 ^a^	7.04 ± 0.67 ^a^	2.31 ± 0.09 ^b^	3.41 ± 0.85 ^a^	0 ± 0 ^a^
0.01 µM C77	6 ± 0.26 ^b^	19.02 ± 0.85 ^c^	3.51 ± 0.19 ^d^	5.46 ± 0.64 ^b^	0 ± 0 ^a^
0.1 µM C77	6.2 ± 0.28 ^b^	23.11 ± 0.63 ^d^	3.19 ± 0.21 ^cd^	5.21 ± 0.79 ^b^	0 ± 0 ^a^
1 µM C77	10.72 ± 0.3 ^c^	10.19 ± 0.73 ^b^	2.59 ± 0.13 ^bc^	7.6 ± 0.77 ^c^	0 ± 0 ^a^
10 µM C77	10.89 ± 0.41 ^c^	13.09 ± 0.95 ^b^	1.03 ± 0.07 ^a^	2.89 ± 0.32 ^a^	38.11 ± 2.35 ^b^

Averages ± SE followed by the same letter in the same column are not significantly different at *p* < 0.05 according to the Kruskal–Wallis H test.

## Data Availability

The raw data supporting the conclusions of this article will be made available by the authors on request.

## Supplementary Materials

The following supporting information can be downloaded at: https://www.mdpi.com/article/10.3390/plants14010108/s1, **Figure S1**: Effect of 1 µM DCP, DCPE, and C77 on the rooting of poplar plants after 2 days (scale bar = 1 mm). The treatments included: (a) Control (hormone-free medium); (b) 1 µM DCP; (c) 1 µM DCPE; (d) 1 µM C77; Figure S2: Effect of 1 µM DCP, DCPE, and C77 on the rooting of poplar plants after 4 days (scale bar = 1 cm). Treatments from left to right: Control (hormone-free medium); 1 µM DCP; 1 µM DCPE; and 1 µM C77; Table S1: Trendline equations and coefficient of determination (R^2^) for different plant parts treated with DCP, DCPE, and C77.
