# Supplementary material for: In Vitro Rooting of Poplar: Effects and Metabolism of Dichlorprop Auxin Ester Prodrugs
Source: Plants (Basel). 2025 Jan 2;14(1):108. doi: 10.3390/plants14010108 (PMC11723099; doi:10.3390/plants14010108)
Supplement: Supplementary file 1 [file plants-14-00108-s001.zip › plants-3403385-supplementary.pdf]

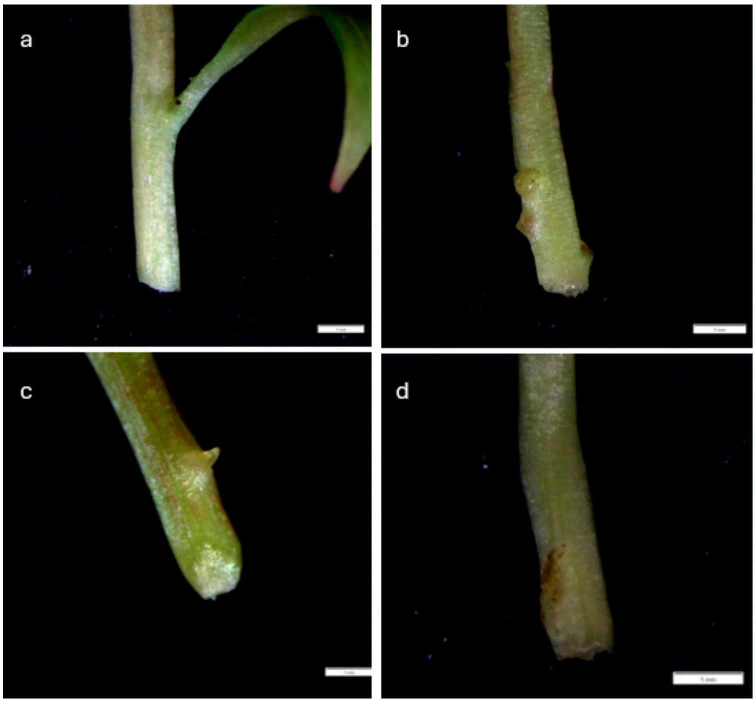

Figure S1. Effect of 1 μM DCP, DCPE, and C77 on the rooting of poplar plants after 2 days (scale bar = 1 mm). The treatments included: (a) control (hormone-free medium); (b) 1 μM DCP; (c) 1 μM DCPE; (d) 1 μM C77.

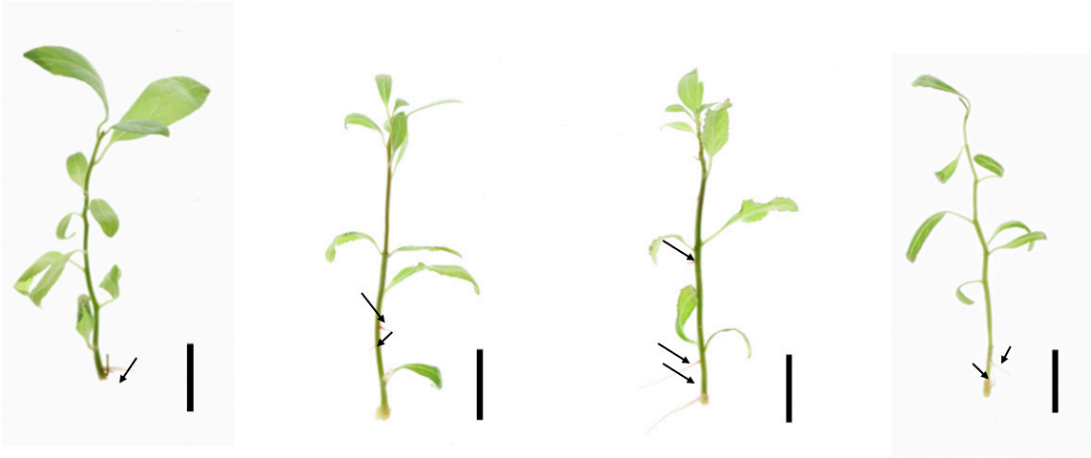

Figure S2. Effect of 1 μM DCP, DCPE, and C77 on the rooting of poplar plants after 4 days (scale bar = 1 cm). Treatments from left to right: Control (hormone-free medium); 1 μM DCP; 1 μM DCPE; and 1 μM C77.

Table S1. Trendline equations and coefficient of determination (R^2) for different plant parts treated with DCP, DCPE and C77.

Plant Section	Treatment Type	Equation of the Trendline	Coefficient of Determination (R^2)
Upper leaves	Plants treated with DCP<br>Plants treated with DCPE	$y = 1.9738x^{-0.822}$<br>$y = -1.278\ln(x) + 3.909$	$R^2 = 0.9936$<br>$R^2 = 0.9676$
Upper stems	Plants treated with DCP<br>Plants treated with DCPE	$y = 2.8281x^{-0.426}$<br>$y = -0.0895x^2 + 0.6773x + 3.15$	$R^2 = 0.9316$<br>$R^2 = 0.9276$
Middle leaves	Plants treated with DCP<br>Plants treated with DCPE	$y = 1.7567x^{-0.56}$<br>$y = -0.1169x^2 + 0.8054x + 2.0217$	$R^2 = 0.9397$<br>$R^2 = 0.9865$
Middle stems	Plants treated with DCP<br>Plants treated with DCPE	$y = 2.7525x^{-0.18}$<br>$y = -0.2935x^2 + 2.5496x - 0.5367$	$R^2 = 0.8775$<br>$R^2 = 0.9771$
Basal leaves	Plants treated with DCP<br>Plants treated with DCPE<br>Plants treated with C77	$y = -0.0263x^2 + 0.041x + 2.6717$<br>$y = -0.1602x^2 + 1.2654x + 1.545$<br>$y = 0.4339\ln(x) + 0.3585$	$R^2 = 0.9998$<br>$R^2 = 0.9955$<br>$R^2 = 0.9667$
Basal stems	Plants treated with DCP<br>Plants treated with DCPE<br>Plants treated with C77	$y = -0.0094x^2 - 0.1123x + 2.77$<br>$y = -0.1892x^2 + 1.8661x + 0.14$<br>$y = 2.879\ln(x) + 3.3607$	$R^2 = 0.998$<br>$R^2 = 1$<br>$R^2 = 0.9645$
